# Author Correction: Active Printed Materials for Complex Self-Evolving Deformations

**DOI:** 10.1038/s41598-018-32403-4

**Published:** 2018-10-22

**Authors:** Dan Raviv, Wei Zhao, Carrie McKnelly, Athina Papadopoulou, Achuta Kadambi, Boxin Shi, Shai Hirsch, Daniel Dikovsky, Michael Zyracki, Carlos Olguin, Ramesh Raskar, Skylar Tibbits

**Affiliations:** 1 Camera Culture Group, Media Lab, Massachusetts Institute of Technology, 75 Amherst St, Cambridge, MA USA; 2 Bio/Nano/Programmable Matter Group, Autodesk Research, Autodesk Inc. Pier 9, San Francisco, CA USA; 3 Self-Assembly Laboratory, Massachusetts Institute of Technology, 265 Massachusetts Ave, Cambridge, MA USA; 4 Stratasys, ltd. Rehovot Science Park, Rehovot, Israel; 5 Singapore University of Technology and Design, 20 Dover Dr, Singapore, Singapore; 6 Bio/Nano/Programmable Matter Group, Autodesk Research, Autodesk Software Co.,Ltd. 399 Pu Dian Road, Shanghai, Pudong District, Shanghai, P. R. China

Correction to: *Scientific Reports* 10.1038/srep07422, published online 18 December 2014

A supplementary file containing Appendices A and B was omitted from this Article and is provided below.

## Electronic supplementary material

Supplemental Data

