# Supplementary material for: Author Correction: Active Printed Materials for Complex Self-Evolving Deformations
Source: Sci Rep. 2018 Oct 22;8:15485. doi: 10.1038/s41598-018-32403-4 (PMC6197236; doi:10.1038/s41598-018-32403-4)
Supplement: Supplementary file 1 — Supplemental Data [file 41598_2018_32403_MOESM1_ESM.pdf]

Active Printed Materials for Complex Self-Evolving Deformations

Dan Raviv[1], Wei Zhao[6], Carrie McKnelly[3], Athina Papadopoulou[3], Achuta Kadambi[1], Boxin Shi[1,5], Shai Hirsch[4], Daniel Dikovsky[4], Michael Zyracki[2], Carlos Olguin[2], Ramesh Raskar[1] & Skylar Tibbits[3]

[1]Camera Culture Group, Media Lab, Massachusetts Institute of Technology, 75 Amherst St, Cambridge, MA, [2]Bio/Nano/Programmable Matter Group, Autodesk Research, Autodesk Inc. Pier 9, San Francisco, CA, [3]Self-Assembly Laboratory, Massachusetts Institute of Technology, 265 Massachusetts Ave, Cambridge, MA, [4]Stratasys, ltd. Rehovot Science Park, Rehovot, Israel, [5]Singapore University of Technology and Design, 20 Dover Dr, Singapore, [6]Bio/Nano/Programmable Matter Group, Autodesk Research, Autodesk Software Co.,Ltd. 399 Pu Dian Road, Shanghai, Pudong District, Shanghai PRC.

Appendix A: Spring-mass modeling

A spring-mass system consists of a set of mass points that are connected by springs where the position, velocity and acceleration over time can be calculated from elementary physics. Specifically, the force on particle x_i due to its connectivity to particle j, becomes

$$f_s^i = k_s \frac{x_j - x_i}{|x_j - x_i|} l \,, \qquad (1)$$

where $x_k \in \mathbb{R}^3$ are the coordinates of a particle in space, k_s is the stiffness coefficient of the spring, and given the final rest length between particles x_i and x_j is l_0, then $l = |x_i - x_j| - l_0$.

A key challenge in applying the spring-mass models to our problem lies in the calibration where the system must imitate the true behavior of the deformable material. For flexibility and ease of configuration, we opt for a spring-mass system that combines both the rigid and deformable elements of the simulated system. In particular, we use the same stiffness coefficient for both rigid and deformable elements while forcing rigidity by introducing angular (non-linear) constraints. In order to imitate the true behavior of the expanding materials the forces are obtained by changing the rest lengths in between the initial and final state in several time intervals. This approach cancels out the jiggling phenomena of a spring-mass system without the need to carefully calibrate the spring's stiffness coefficient.

We follow the conventional approach where damping is proportional to the velocity. Meaning,

$$f_d^i = k_d(v_j - v_i)\frac{x_j - x_i}{|x_j - x_i|}, \qquad (2)$$

where $v_k \in \mathbb{R}^3$ is the spatial velocity and k_d is the damping coefficient. In an ideal spring-mass damper system the damping ratio is defined as $\mathcal{E} = \frac{k_d}{2}\sqrt{k_s m}$, where m is the mass. Choosing the correct damping is done by setting the damping ratio to 1.

The stiffness coefficient is approximated by moment equilibrium (see Figure 3B), as $0.5FR = F'L$. Assuming that the acceleration of rigid bar is a and it is folds α degrees in time t, we have $L\alpha \approx 0.5at^2$. Because $F = k_s(l - l_0)$, and $F' = ma$, we readily have that $k_s = \frac{4mL\alpha}{(l-l_0)Rt^2}$. For example, if we set $m = 10$ $(gram)$, $L = 0.015$ $(meter)$, α=0.785, $l - l_0 = 0.002$ $(meter)$, $r = 0.005$ $(meter)$, $t = 10$ $(second)$, then $k_s = 471$ (N/m).

Appendix B: Simulator

The Nucleus simulator was first introduced in Autodesk MAYA 8.5 and can be found as a standalone library. We have used Nucleus in this paper as part of *Project Cyborg,* a new design platform in development from Autodesk. The advantage of Nucleus is with its simplicity to model geometric constraints on different elements, referred to as simplical complex. There are three key constraints in Nucleus that are relevant to our simulation: 1) Length between edges (1-simplices), 2) angle between each pair of edges (1-simplices), and 3) angle along an edge (1-simplices) connecting two 2-simplices. Both the stretching and folding primitives can be described using these constraints. All of the constraints are written as functions of the spatial location of the elements in space, and a non-linear solver is used for the deformation.

One major drawback of the spring-mass system is that the system can over compensate for the movement, causing the simulation to act in a non-natural way. Such phenomena occur when the system converges to a local minimum but the springs go from contraction to expansion and vise versa. To overcome this problem in hand, we do not allow for large deviations by setting the time step in Nucleus to 1/24 second. We built a wrapper on-top of Nucleus which forces a gradual change of the springs rest length for all primitives as the simulation progresses.
